# MicroRNA-182 downregulates metastasis suppressor 1 and contributes to metastasis of hepatocellular carcinoma

**DOI:** 10.1186/1471-2407-12-227

**Published:** 2012-06-08

**Authors:** Jian Wang, Jingwu Li, Junling Shen, Chen Wang, Lili Yang, Xinwei Zhang

**Affiliations:** 1 Department of 4th Abdominal Oncology, Cancer Hospital and Institute of Tianjin Medical University, Tianjin, 300060, China; 2 Department of Abdominal Oncology, Tangshan People's Hospital, Tangshan, 063001, China; 3 Hebei Medical University, Shijiazhuang, 050017, China; 4 Qingdao Agricultural University, Qingdao, 266109, China

**Keywords:** Hepatocellular carcinoma, *miR-182*, Metastasis suppressor 1, Metastasis

## Abstract

**Background:**

*miR-182* is one of the most significantly up-regulated miRNAs in hepatocellular carcinoma (HCC). Metastasis suppressor 1 (*MTSS1*), one target gene of *miR-182*, plays an important role in the metastasis of cancers. However, it remains unclear what role does function and mechanism of *miR-182* and *MTSS1*play in HCC.

**Methods:**

*miR-182* expression was tested in 86 cases of paired HCC and normal tissues by real-time PCR and the relationships between *miR-182* expression and clinicopathological parameters were analyzed. The expression of *MTSS1* was evaluated by immunohistochemistry and western blot in the above tissues and its correlation with *miR-182* expression was analyzed. Moreover, western blot and invasion assays were performed after transfection of *pre*-*miR-182* or *anti*-*miR-182* to HCC cell lines. In addition, luciferase assays was performed to confirm the regulation of *miR-182* on *MTSS1*.

**Results:**

Compared with normal tissue, *miR-182* was up-regulated and *MTSS1* was down-regulated in HCC tissues. Moreover, the over-expression of *miR-182* was correlated with intrahepatic metastasis (*p* = 0.034) and poor prognosis (*p* = 0.039) of HCC patients. There was a negative correlation between *miR-182* and *MTSS1* expression in both HCC tissues (*r* = −0.673, *p* < 0.01) and HCC cell lines (*r* = −0.931, *p* = 0.021). Furthermore, the up-regulation of *miR-182* resulted in the down-regulation of *MTSS1* and increased invasive potential of HUH-1, and reverse results were also confirmed when the expression of *miR-182* was inhibited. In addition, the results of the luciferase assay demonstrated the targeted regulation of *miR-182* on *MTSS1*.

**Conclusions:**

*miR-182* could promote metastasis of HCC and inhibit the expression of *MTSS1*. *miR-182* and *MTSS1* are potential prognostic markers and/or therapeutic targets in HCC.

## Background

Hepatocellular carcinoma (HCC), one of the most notoriously invasive cancers, is among the top 10 most prevalent cancers worldwide, accounting for ~600,000 deaths annually [1,2]. At present, surgical resection/liver transplantation is the only treatment modality to confer survival benefit in HCC patients, and the overall 5-year survival rate for HCC patients is less than 5% [3]. The most important reason leading to poor prognosis is intra hepatic metastasis [4]. It is thus necessary to elucidate the molecular mechanisms underlying HCC metastasis and identify novel therapeutic targets.

Recently, it has been manifested that the deregulation or dysfunction of miRNAs is involved in cancer development and related to clinical outcomes of cancer patients including HCC [5-12]. Yu, et al reported *miR-182* was one of the most significantly up-regulated miRNA in HCC patients [13]. Aberrant *miR-182* expression promotes melanoma metastasis by repressing *FOXO3* and microphthalmia-associated transcription factor [14,15], which indicates that *miR-182* may promote the metastasis of HCC through targeting on some genes. In both websites Target scan and Pictar, we found hundreds of target genes regulated by *miR-182*. Among those genes with highly conserved binding sites, metastasis suppressor 1 (*MTSS1*) brought us lots of concern as it has been demonstrated to have prognostic value and anti-metastatic properties in breast cancer [16] and gastric cancer [17].

We then tested the expressions of *MTSS1* and *miR-182* in paired normal liver and HCC tissues. Statistics analysis demonstrated the negative correlation between *miR-182* and *MTSS1* and the important clinicopathological significance of *miR-182* in HCC patients. Experiments in vitro further confirmed that *miR-182* can promote the metastasis of HCC cell lines and down-regulate *MTSS1*, which further elucidate the metastatic mechanism of HCC and may suggest novel findings for targeted treatment.

## Methods

### Patients and samples

Informed consent was obtained from all the patients for the collection of liver specimens, and the study protocol was approved by the Ethics Committee of Tianjin Medical University. The investigations were conducted according to the Declaration of Helsinki Principles. The clinical pathological data were collected as described in our earlier study [2]. Eighty-six primary HCC patients treated in Cancer Hospital of Tianjin Medical University between 2004 and 2007 were selected according to the following criteria: (1) The diagnosis of HCC was confirmed by pathology; (2) No preoperational chemotherapy or TAE were performed; (3) All of the samples were from the hepatectomy for the first time; (4) Incisal margins were negative; and (5) Clinicopathologic data of the cases could be collected. Among the 86 patients, there were 67 men (77.9%) and 19 women (22.1%). The mean age at diagnosis was 50.7 ± 9.7 years, ranging from 29 to 78 years. HBV was positive in 72 patients (83.7%). The percentage of AFP (>100 ng/ml) was 80.2%. Moreover, one tumor was detected in 79.1% (68/86) patients and multiple tumors (≥2) were found in 20.9% (18/86) patients with totally 29 metastatic leisions. The average tumor size was 5.8 ± 2.7 cm (0.4-16 cm). Histologically, 32.6% (28/86), 46.5% (40/86) and 20.9% (18/86) tumors were grade 1, 2 and 3, respectively. No chemotherapy was performed after radical resection. Patients were followed-up at the outpatient clinic with measurement of the serum alpha-fetoprotein level and hepatic ultrasonography every 2–4 months from the date of initial treatment. The mean time of follow-up was 28.3 months (range 3– 56 months). When recurrence was suspected, further evaluations were performed by abdominal computed tomography (CT) scan, if necessary, by ultrasound-guided biopsy to confirm the diagnosis. Recurrence was observed in 46.5% (40/86) patients. HCC and non-neoplastic tissues were collected and stored at −80°C until analysis. For every frozen tumor tissue, we cut frozen slide and did HE staining and evaluated the percentage of tumor cells. The percentage of tumor cells was about 90%. In addition, paraffin-embedded HCC tissues were also collected.

### RNA extraction and quantitative RT-PCR for *miR-182*

Total RNA, including miRNA, was extracted using TRIzol reagent (Invitrogen, Carlsbad, CA, USA) according to the manufacturer’s instructions. Total RNA was reversely transcribed using the corresponding RT Primer and the TaqMan MicroRNA Reverse Transcription Kit (Applied Biosystems). The expression of *miR-182* and its control RNU44 were detected using TaqMan miRNA assay system (Applied Biosystems, Foster City, CA, USA). The median miRNA intensity value of 86 patient samples was used as the threshold, and patients were divided into two groups (below median, group low *miR-182* and above median, group high *miR-182*) according to the expression of *miR-182*.

### Immunohistochemistry staining and evaluation for *MTSS1*

Immunohistochemistry (IHC) was used to detect *MTSS1* expression in paraffin-embedded HCC tissues. Five-μm sections of paraffin-embedded HCC tissue were baked at 65°C for 2 h, followed by deparaffinization using standard procedures. After antigen retrieval, *MTSS1* antibody (Cell Signaling Technology, Inc. Danvers, MA, USA) was applied to slides, followed by the secondary antibody conjugated with horseradish peroxidase. Signals were revealed by using the Histostain Plus kit (Invitrogen, Grand Island, NY, USA) according to the manufacturer's instruction. 3, 3-Diaminobenzidine (DAB) was used as a chromogen. The sections were counter-stained with hematoxylin. We prepared a negative control by substituting PBS for the antibody.

*MTSS1* protein expression was evaluated by two pathologists. *MTSS1*-positive samples were defined as those with brown staining in the cytoplasm. The results of *MTSS1* immunohistochemical analysis were estimated with semi-quantity method. The staining intensity was graded on a scale from 0 to 3 (0 for no staining, 1 for weak immunoreactivity, 2 for moderate immunoreactivity, and 3 for strong immunoreactivity) The percentage of immunoreactivity was scored on a scale from 0 to 4 (0, no positive cells; 1, <25% of cells positive; 2, 25%–50% of cells positive; 3, 50– 75% of cells positive; and 4, >75% cells positive). Finally, a total score (negative: 0; weak: 1–2; medium: 3–5; strong: 6–7) was obtained by adding the scores of staining intensity and percentage positivity.

### Western blot for *MTSS1*

Cell lysates were harvested with 2% sodium dodecyl sulfate (SDS)-125 mM Tris/HCl (pH 7.4). Cell lysates (25–30 ug of protein) were resolved in Tris/glycine SDS/PAGE gels and transferred to PVDF membranes. Membranes were probed with primary antibodies overnight at 4°C and incubated with horseradish-peroxidase-coupled secondary antibodies (Santa Cruz Biotechnology, Santa Cruz, CA, USA). The background was subtracted, and the signals of the detected bands were normalized to the amount of loading control β-actin (Cell Signaling Technology, Inc. Danvers, MA, USA) band. The protein levels were quantified using ImageJ software (National Institute of Mental Health, Bethesda, MD, USA. http://rsb.info.nih.gov/ij).

### Cell culture and transfection

Human HCC cell lines HLE, HLF, HepG2, Hep3B and HUH-1 were obtained from American Type Culture Collection (Manassas, VA, USA) and cultured in DMEM (Invitrogen) except HepG2 (MEM) supplemented with heat-inactivated 10% fetal bovine serum (Invitrogen) at 37°C in a humidified incubator containing 5% CO_2_.

For transfection, 2 × 10^5^ HLF or HUH-1 cells were seeded into each well of a 6-well plate and incubated overnight, then the cells were transfected with Pre-miR miRNA Precursor Molecule *pre-182* (*pre-miR-182*) and anti-miR miRNA inhibitor *anti-182* (*anti-miR-182*) (Applied Biosystems) at a final concentration of 100 nM using the Lipofectamine 2000 transfection reagent (Invitrogen, Carlsbad, CA, USA), according to the manufacturer’s instructions. The specificity of the transfection was verified using the Pre-miR miRNA Precursor Molecule Negative Control #1 (control pre-miR) and Anti-miR miRNA Inhibitors Negative Control #1 (control anti-miR) (Applied Biosystems). The expression levels of *miR-182* and *MTSS1* were quantified 24 h after transfection, and the cells were used for western blot analysis.

### 3’ UTR luciferase reporter assay

The human *MTSS1* 3’ UTR luciferase reporter construct (*MTSS1*-3’UTR WT) was generated by cloning *MTSS1* mRNA 3’UTR sequence into downstream of pMIR-Report construct (Ambion, Foster City, CA, USA). The *MTSS1* 3’ UTR sequence was generated by PCR using primer *MTSS1* 3’UTR F SpeI: 5’-AAACTAGTTGATTTTTCTGAAGGT GCCAAATTCCATTTAA-3’ and primer *MTSS1* 3’UTR R SacI: 5’–GGGAGCTCTTTGGCAACATTTTATTTATTCA-3’. The *miR-182* target site-mutation *MTSS1* 3’ UTR luciferase reporter 1 (*MTSS1*-3’UTR mutation 1) construct was generated by employing direct-site mutagenesis using mutation primers which mutate the *miR-182* binding site from TCTGAAGGTGCCAA to GATGAAGGTCGGTA. *miR-182* target site-mutation *MTSS1* 3’ UTR luciferase reporter (*MTSS1*-3’UTR mutation 2) was mutated from TTGCCAA to TAACGCT in the *miR-182* binding site. *MTSS1*-3’UTR mutation 1, 2 was mutated in these two *miR-182* binding sites.

HUH-1 cells were co-transfected with *miR-182* plasmid and wild-type or mutant *MTSS1* 3’ UTR luciferase reporter construct and luciferase activities were measured using the Dual-Glo Luciferase. Data were normalized by dividing Firefly luciferase activity with that of Renilla luciferase.

### In-vitro invasion assays

HLF and HUH-1 cell invasion assays were performed using 24-well Matrigel Invasion Chambers (BD Biosciences, CA, USA). The lower chambers were filled with 0.75 ml of DMEM medium containing 10% fetal bovine serum (FBS). A cell suspension of 2 × 10^5^ in 0.5 ml DMEM medium was added into each well of the upper chamber. After the cells were incubated for 24 h at 37°C in a humidified incubator with 5% CO_2_, The invasive cells attached to the lower surface of the membrane insert were fixed in 10% formalin at room temperature for 5 min and stained with 0.05% crystal violet. The non-invading cells that remained on the upper surface of the membrane were removed by scraping. The number of invasive cells on the lower surface of the membrane was then counted under a microscope.

### Statistical analysis

Differences in *MTSS1* immunohistochemical staining between groups were compared using chi-square or Fisher exact tests in human samples. The correlation between *MTSS1* expression and *miR-182* was evaluated by calculating the Spearman rank correlation coefficient. Moreover, mean ± SD of clinicopathological variables were calculated, and differences in the means were analyzed using one-way analysis of variance or Student’s t test. We also used the Kaplan-Meier method and the log-rank test in univariate survival analysis, and we used the Cox proportional hazards regression model in our multivariate analysis. SPSS version 16.0 (IBM) was used to perform our statistical analysis. Two-tailed *P* values < 0.05 were considered statistically significant.

## Results

### The expression of *miR-182* and its correlation with clinical-pathological features

**Figure 1:**
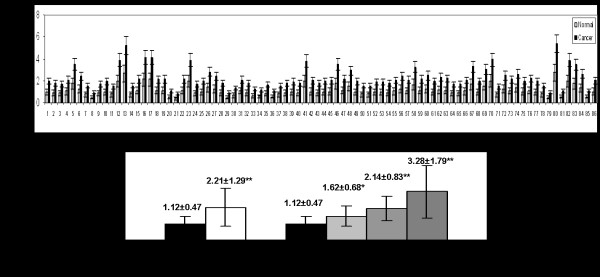
**Expression of*miR-182*in normal and tumor tissues of 86 HCC patients.** Relative expression of *miR-182* was detected by real time PCR in every paired normal and HCC tissues **(A)**. The relative expression of *miR-182* in HCC tissues (n = 86, 2.21 ± 1.29) was significantly higher than that of normal liver tissues (1.12 ± 0.47) **(B)**. The relative level of *miR-182* in HCC of grade 1, grade 2 and grade 3 were 1.62 ± 0.68, 2.14 ± 0.83 and 3.28 ± 1.79, repectively, which were also significantly higher than that of normal tissues. * *p* < 0.05; ** *p* < 0.01.

To investigate the role of *miR-182* in HCC development, we tested the expression of *miR-182* in 86 HCC and matched non-neoplastic tissues (Figure 1A). The relative expression of *miR-182* in HCC samples (2.21 ± 1.29) was significantly higher than that of matched normal tissues (1.12 ± 0.47) (*p* < 0.01) (Figure 1B). Hence, we considered the up-regulation of *miR-182* may contribute to HCC tumorigenesis. Furthermore, the relative level of *miR-182* in poorly differentiated HCC (3.28 ± 1.79) was almost one time higher than that in well (1.62 ± 0.68) and medium differentiated cases (2.14 ± 0.83) (Figure 1B), which suggested that *miR-182* might also correlate with the progress of HCC.

For better understanding the potential role of *miR-182* in HCC progression, we analyzed its correlation with some clinicopathological variables including age, sex, HBV infection, AFP, tumor number, tumor size, expression of *MTSS1*, histological grade, portal vein invasion and recurrent time (Table 1). Based on the median value (1.92) of *miR-182* expression, all patients were divided into two groups including group with low expression of *miR-182* and group with high expression of *miR-182*. Intra-hepatic metastasis (tumor number ≥2, *p* = 0.034) and higher recurrence (*p* = 0.031) tended to occur in the patients with high expression of *miR-182*. Though the *p* values did not reach statistical significance, the patients with high expression of *miR-182* had a tendency to undergo occur portal vein invasion (*p* = 0.096) (Table 1).

**Table 1:** The relationships between miR-182 expression and clinicopathologic features

**Variables**	**Low miR-182 expression (n = 43)**	**High miR-182 expression (n = 43)**	***p***
Age (<51 years *vs* ≥51 years)	21:22	25:18	0.387
Gender (male *vs* female)	35:8	32:11	0.436
HBV (positive *vs* negative)	37:6	35:8	0.341
AFP(<100 ng/ml *vs* ≥100 ng/ml)	10:33	7:36	0.417
Tumor number (n < 2 *vs* n ≥ 2)	38:5	30:13	0.034
Tumor size (<5 cm *vs* ≥5 cm )	16:27	19:24	0.510
MTSS1 (positive *vs* negative)	21:22	16:27	0.115
Tumor grade (1 *vs* 2, 3)	16:27	12:31	0.174
Portal vein invasion (no *vs* yes)	34:9	27:16	0.096
Recurrent (no *vs* yes)	28:15	18:25	0.031

*AFP*: α-fetoprotein.

**Figure 2:**
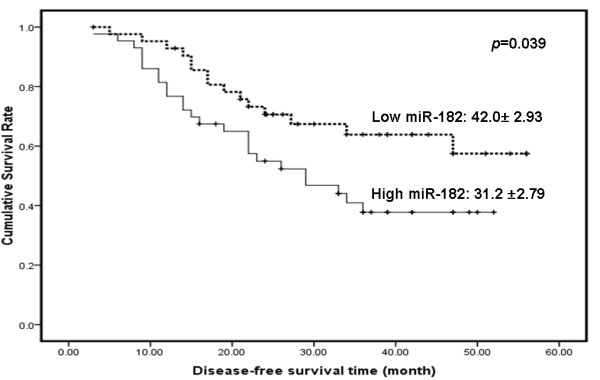
**Univariate analysis of disease-free survival for*miR-182*expression in 86 HCC patients.** The median value (1.92) of miR-182 level was chosen as the cutoff point for separating miR-182 low-expression tumors (n = 43) from miR-182 high-expression cases (n = 43). *p*-value was shown with the use of log-rank test in SPSS 11.5. *p* = 0.039 (Kaplan–Meier log-rank survival analyses).

The recurrent percentage is 46.5% (40/86) for all patients. The median disease-free survival time in group with low *miR-182* and group with high *miR-182* was 27.0 months and 24.0 months, respectively. The Kaplan–Meier method revealed that higher *miR-182* expression level correlated with significantly reduced disease-free survival (42.0 ± 2.93 months in group with low *miR-182* versus 31.2 ±2.79 months in group with high *miR-182*, *p* = 0.039) (Figure 2). Multivariate survival analysis revealed that multiple tumors (*p* = 0.023) and high expression of *miR-182* (*p* = 0.022) were significantly correlated with the poor prognosis of HCC patients (Table 2). The result further indicated the importance of *miR-182* up-regulation in HCC development. 

**Table 2:** Multivariate Cox regression analysis for disease-free survival in 86 HCC patients

**Variables**	**HR (95% CI)**	***p***
Age (≥51 years *vs* <51 years)	0.762 (0.683-2.701)	0.383
Gender (female *vs* male)	0.525 (0.612-2.905)	0.469
HBV (positive *vs* negative)	0.078 (0.365-2.129)	0.780
AFP (≥100 ng/ml *vs* <100 ng/ml)	0.884 (0.553-5.397)	0.347
Tumor number (n ≥ 2 *vs* n < 2)	5.132 (1.119-4.727)	0.023
Tumor size (≥5 cm *vs* <5 cm)	0.497 (0.392-1.554)	0.481
Tumor grade (2,3 *vs* 1)	0.251 (0.571-2.574)	0.616
Portal vein invasion (yes *vs* no)	2.824 (0.901-3.872)	0.093
miR-182expression (high *vs* low)	4.560 (1.073-5.201)	0.033
MTSS1 (negative *vs* positive)	3.109 (0.225-1.082)	0.078

### The expression of *MTSS1* is down-regulated and negatively correlated with *miR-182* in HCC

**Figure 3:**
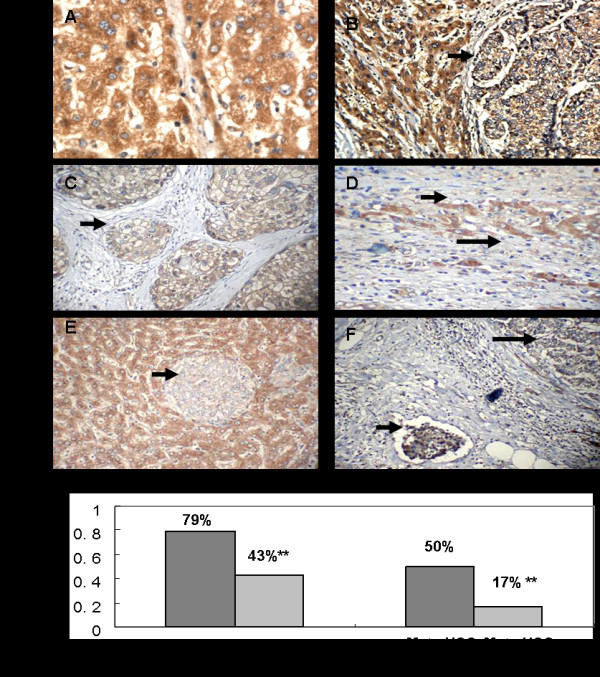
**Immunohistochemistry staining for MTSS1 in HCC and paired normal tissues (200×).A:** Normal (non-neoplastic) liver tissue; **B** and **C:** positive expression of MTSS1 in well-(B) and moderate-(C) differentiated tumors. **D:** poorly differentiated cancer cells without MTSS1 expression (long arrow) infiltrated into normal liver cell panel with MTSS1 high expression (short arrow); **E:** MTSS1 expression was lower in the tumor foci (arrow) compared with around non-neoplastic liver tissue; **F:** MTSS1 expression is low in tumor thromboses (short arrow) and primary HCC (long arrow). **G:** The positive rate of MTSS1 in HCC tissues (43%) was significantly lower than that of normal tissues (79%). Furthermore, the positive rate (17%) in patients with metastasis (tumor number ≥2) was significantly lower than that of cases without metastasis (50%). ** p < 0.01.

*MTSS1* protein expression was tested with IHC in HCC and paired normal tissues (for some cases, there are tumor and adjacent normal tissue in the same slide). *MTSS1* was positive in the cytoplasm of tumor and normal liver cells. *MTSS1* was often highly expressed in normal tissue (Figure 3 A, B and E), while drastically reduced *MTSS1* expression was shown in the tumor cells (Figure 3 B, D and E). Totally, MTSS1 was positive in 79% (68/86) of normal tissue and in 43% (37/86) of HCCs (43%). The rate of MTSS1 positive case in HCC was significantly lower than that in paired normal tissue (*p* < 0.001, Figure 3G). Among the 37 cases with MTSS1 positive expression, 13 (35%), 17 (46%) and 7 (19%) cases showed weak, mederate and strong expression of MTSS1, respectively. Moreover, no difference of MTSS1 expression was found among the multiple lesions in the same patient. The MTSS1 positive rate in metastatic HCC (17%, 3/18) was significantly lower than that in non-metastatic HCC (50%, 34/68) (*p* < 0.001, Figure 3G). In addition, the tumor thrombus in small hepatic vein also showed low expression of MTSS1 (Figure 3 F).

**Figure 4:**
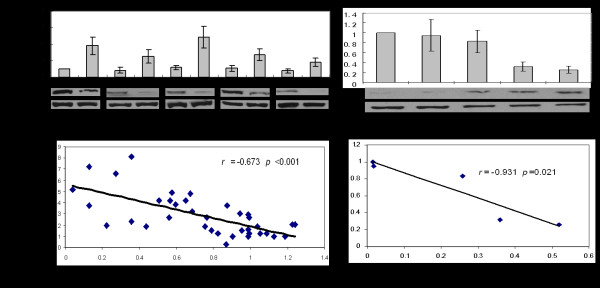
**Correlation between miR-182 and MTSS1 expression in HCC tissues and cell lines.A:** Relative expression of miR-182 was detected by real time RT-PCR and MTSS1 expression was tested with Western blot and quantified with ImageJ software in 5 paired HCC and normal tissues. **B:** There was a negative correlation between miR-182 and MTSS1 expression (n = 37). **C:** The expression of miR-182 is high and MTSS1 expression is low in HLF and HLE, whereas Hep-G2 and HUH-1 showed low expression of miR-182 and high expression of MTSS1. **D:** The expression of MTSS1 was negatively correlated with miR-182 expression in HCC cell lines. N: normal tissue; T: tumor.

For the *MTSS1* positive cases tested by IHC, the examination of Western Blot were further performed and the expressions were quantified (Figure 4 A). Negative correlation between the expression of *miR-182* and that of *MTSS1* in HCC was indicated in Figure 4 B (*r* = −0.673; *p* < 0.01), which suggested *MTSS1* maybe one important functional protein contributing to the oncogenic role of *miR-182*. Meanwhile, the negative correlation between *miR-182* and *MTSS1* expression were also found in HCC cell lines (*r* = −0.931, *p* = 0.021) (Figure 4 C and D).

### *miR-182* promotes invasion and inhibits *MTSS1*

**Figure 5:**
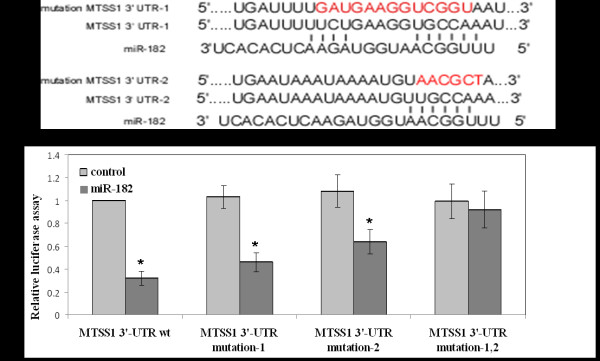
**miR-182 directly targets human MTSS1. A:** 3’-UTR region of MTSS1 mRNA is partially complementary to miR-182. Target-Scan and Pictar analysis revealed two miR-182 binding sites in MTSS1 UTR sequence. **B:** Effect of miR-182 on the luciferase activity of Luc-MTSS1-3’UTR and Luc- MTSS1-3’UTR mutation. The assay was done in HUH-1 cells as described in Materials and Methods. Renilla and firefly luciferase activities were measured with the Dual-Luciferase Reporter system (Promega) 24 h after transfection. Firefly luciferase activity was normalized to Renilla luciferase expression for each sample. Each experiment was performed in triplicate. Data are shown as mean ± s.d. *p < 0.01.

Next, we sought to investigate the molecular mechanism responsible for the oncogene effect of *miR-182* on HCC observed above. As miRNAs function mainly through inhibiting their target mRNAs by binding to the 3’ UTR, we searched the putative target genes of *miR-182* in Target Scan and Pictar. In both websites, 841 and 702 conserved targets were found, respectively. Among those targets, human *MTSS1*, known to have critical roles in the inhibition of cancer metastasis, contained two putative conserved *miR-182* binding sites with high context scores (Figure 5A). To verify whether *MTSS1* was a direct target of *miR-182*, a dual-luciferase reporter system was used by co-transfection of *miR-182* and a luciferase reporter plasmid containing the 3’ UTR of human *MTSS1* into HUH-1. As shown in Figure 5B, the luciferase activity was significantly inhibited by *miR-182* co-transfection, mutation either of the two *miR-182* binding site, while *miR-182* failed to inhibit the expression of luciferase construct with both binding sites mutated, suggesting that *miR-182* could directly target on the 3’ UTR of *MTSS1*.

**Figure 6:**
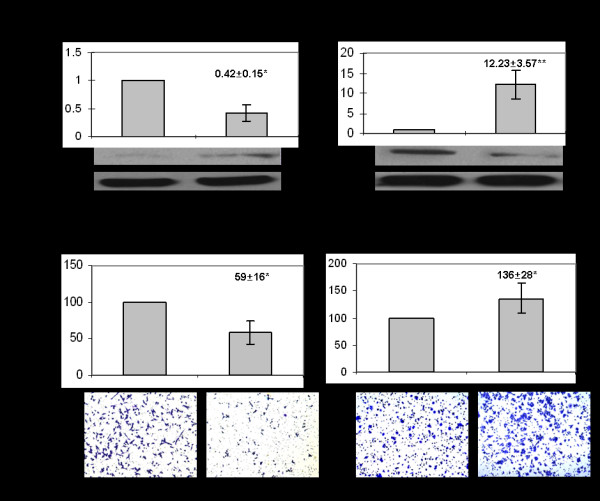
**miR-182 modulated the expression of MTSS1 and the cells invasive potential of HLF and HUH-1.A:** the expression of MTSS1 was up-regulated in HLF and down-regulated in HUH-1 after transfection of anti-miR-182 and pre-miR-182, respectively. Cell invasion assay in HLF and HUH-1 cells were first transfected with anti-mir-182 and pre-mir-182, respectively or negative control and then subject to matrigel chamber assays, as described in Materials and Methods. After 24 h, invasion cells were counted after staining with crystal violet. *p < 0.05, **p < 0.01.

As one target gene of *miR-182* demonstrated above, the expression of *MTSS1* was down-regulated in HUH-1 with transfected *miR-182* and up-regulated in HLF with transfected anti-*miR-182* (Figure 6 A). An *in vitro* invasion assay indicated that the relative invasiveness of HLF transfected with *anti-miR-182* was specifically reduced by approximately 41% (*p* < 0.05) and the relative invasiveness cells of HUH-1 transfected with *miR-182* was increased by approximately 36% (*p* < 0.05) (Figure 6B). The result in *vitro* further demonstrated that miR-182 could promote metastasis of HCC and inhibited the expression of *MTSS1*.

## Discussions

Up-regulation of *miR-182* was suggested to exist in a large part of HCC tissues [15]. In our HCC cases with complete clinical data, we also found the up-regulation of *miR-182* and its up-regulation was significantly associated with intrahepatic metastasis (tumor number ≥ 2) and early recurrence, which is an important clinical determinant for the prognosis of HCC patients. Up-regulation of *miR-182* was further suggested to correlate with reduced disease-free survival of HCC patients. Hence, determination of *miR-182* expression level in HCC tissues may be a novel approach to predict and identify the prognosis of HCC patients.

Although miRNA profile did reveal very prospective features in cancer, the functions and real targets of miRNAs were largely unknown. The predicted targets of the majority of microRNAs based on sequence homology remained to be comprehensively validated by *in vitro* and *in vivo* experiments. Target scan and Pictar showed metastasis suppressor 1 (*MTSS1*) is one important target of *miR-182* with a high context score. Meanwhile, we found its expression in HCC decreased significantly compared to that of adjacent normal tissue and negatively correlated with the expression of *miR-182*, which indicated *MTSS1* maybe the regulation target of *miR-182*.

*MTSS1*, also known as *MIM* (missing in metastasis), was originally identified by Lee et al. [18] as a potential metastasis suppressor gene that was present in non-metastatic bladder cancer cell lines, but was not expressed in a metastatic bladder cancer cell line [19]. This gene, mapped to human chromosome 8q24.1, encodes a 5.3 kb mRNA and a polypeptide predicted to be an actin-binding protein of 356 amino acids with homology to the WASp (Wiscott-Aldrich Syndrome protein) family [20]. Functional analyses of *MTSS1* have shown that *MTSS1* induced actin-rich protrusions resembling microspikes and lamellipodia at the plasma membrane and promoted disassembly of actin stress fibres [21]. Actin filament assembly is associated with cytoskeletal structure organization and many forms of cell motility [22]. These data have suggested that *MTSS1* protein may be important in regulating cytoskeletal dynamics, and as a consequence it would play a potential role in the invasion and metastatic behavior of cancer cells. Therefore, the down-regulation of *MTSS1* potentiated by the up-regulation of *miR-182* may further aggravate the epigenetic changes in HCC. We then focused on the mechanisms that whether the up-regulation of *miR-182* mediates the inhibition of *MTSS1* and induced epigenetic alterations in HCC pathogenesis.

*miR-182* can bind to *MTSS1* at two conserved sites with a high context score. Our luciferase assay in HCC cell lines demonstrated *MTSS1* can be regulated directly by *miR-182*. The interesting results in HCC cell lines is that cells with high invasive ability showed higher expression level of *miR-182* than those with low invasive potential, which is inversely related with the expression of *MTSS1*. Analyses on human samples reinforced the relevance of *miR-182* regulation on *MTSS1* in HCC by revealing an inverse correlation between their expressions. Considering the characteristic heterogeneity of HCC and that *MTSS1* is regulated by additional mechanisms, a statistically significant association with *miR-182* is especially remarkable. The ability of *MTSS1* over-expression to counteract *miR-182*’s pro-invasion effects unequivocally shows the importance of this inverse relationship in HCC metastasis. The functional analysis of *miR-182* together with *MTSS1* in animal models will particularly further evaluate their metastatic role and show us the clinical treatment value for patients with HCC. That would be our future research aim.

Concerning the target of *miR-182*, Miguel and et al. also reported that the microRNA promotes melanoma metastasis by repressing *FOXO3* and microphthalmia-associated transcription factor [13]. Together with our study, it is consistent with current opinions that a single miRNA can target multiple mRNAs, named ‘targetome’, to post-transcriptionally regulate gene expression [23]. Hence, it is probable that we are still far from unveiling the last target of *miR-182*. According to this presumption, interesting future work may be carried out to identify the ‘targetome’ and the entire roles of *miR-182* in cancer development. Another important issue is why *miR-182* is up-regulated in HCC and other cancers [15,24]. The current view suggests that miRNA expression is mainly controlled at the transcriptional level. A large number of transcription regulators that influence the transcription and production of miRNAs have been identified including Myc, E2F, p53, and STAT3 [25-27]. Another possible mechanism for the up-regulation of miRNAs in cancer may result from the amplification of DNA copy number. Such as *miR-182* is one member of a miRNA cluster in a chromosomal locus (7q31-34) frequently amplified in HCC [13], the amplification may cause the up-regulation of *miR- 182*. This is our future’s research field.

## Conclusions

Our study suggests a model of tumor progression in which elevated miR-182 expression and subsequent down-regulation of MTSS1 promotes aggressiveness of HCC. These results suggest that miR-182 and its downstream effectors could prove to be useful prognostic markers and/or therapeutic targets in HCC.

## Competing interests

All authors declare that they have no competing interests.

## Authors’ contributions

JW together with JL conceived of the study, and participated in its design and coordination and helped to draft the manuscript. JS carried out the molecular biological studies and drafted the manuscript. CW collected all the clinicopathological data. LY performed imunohistochemistry assay. XZ participated in the design of the study and performed the statistical analysis. All authors read and approved the final manuscript.

## Pre-publication history

The pre-publication history for this paper can be accessed here:

http://www.biomedcentral.com/1471-2407/12/227/prepub
